# Human *PKD1* sequences form R-loop structures *in vitro*

**DOI:** 10.17912/micropub.biology.001058

**Published:** 2024-02-02

**Authors:** Agata M Parsons, Kemin Su, Maya Daniels, Gerrit J Bouma, Gregory B Vanden Heuvel, Erik D Larson

**Affiliations:** 1 Biomedical Sciences, Western Michigan University Homer Stryker MD School of Medicine, Kalamazoo, Michigan, United States; 2 Investigative Medicine, Western Michigan University Homer Stryker MD School of Medicine, Kalamazoo, Michigan, United States

## Abstract

Autosomal dominant polycystic kidney disease results from the loss of the *PKD1* gene product, polycystin 1. Regulatory mechanisms are unresolved, but an apparent G/C sequence bias in the gene is consistent with co-transcriptional R-loop formation. R-loops regulate gene expression and stability, and they form when newly synthesized RNA extensively pairs with the template DNA to displace the non-template strand. In this study, we tested two human *PKD1* sequences for co-transcriptional R-loop formation in vitro. We observed RNase H-sensitive R-loop formation in intron 1 and 22 sequences, but only in one transcriptional orientation. Therefore, R-loops may participate in *PKD1* expression or stability.

**Figure 1. R-loop formation from PKD1 sequences:**
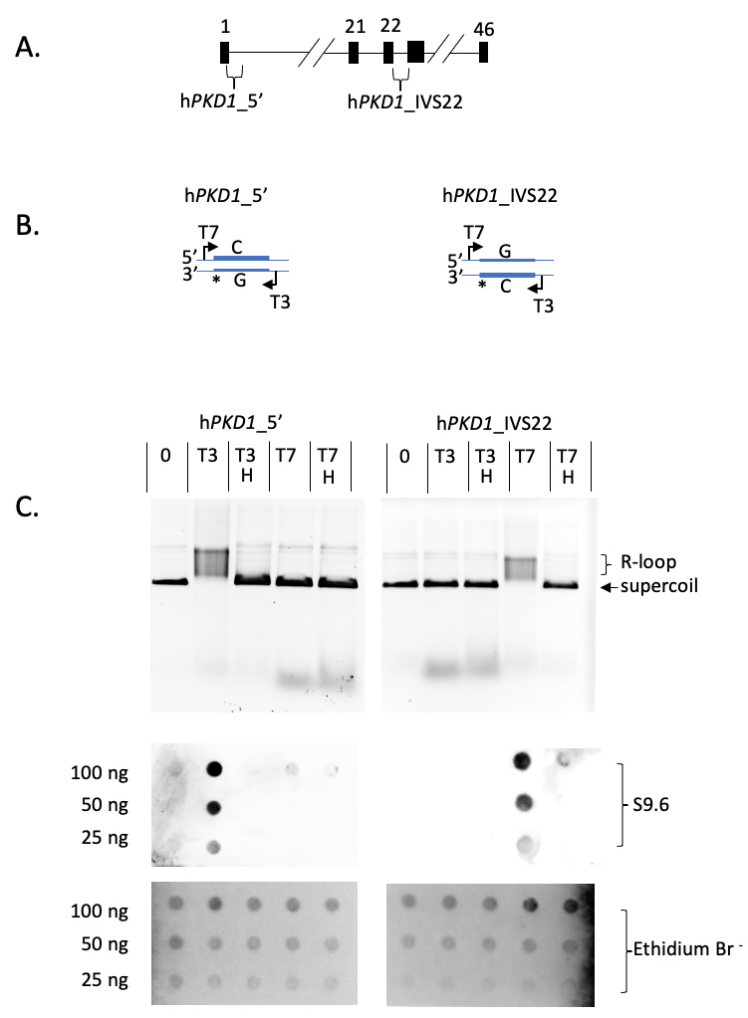
A) C-rich sequences in *PKD1* were selected from the 5’ region of the gene and from intron 22 and synthesized. B) Sequences were placed in between the T7 and T3 promoters of pBluescript II SK ^+^ . The sense strand of *PKD1* is indicated with an asterisk and the C-rich strand by a thick line. As diagramed, T7 transcription templates on the bottom strand, T3 on the top strand C) Top, transcription reactions resolved by agarose electrophoresis, ethidium bromide stained, and UV imaged. Plasmid h *PKD1_5’* (left) or h *PKD1-IVS22* (right) was treated with no polymerase (0), T3, or T7 polymerase (T3, T7). All reactions were post-treated with RNase A, reactions treated with RNase H are indicated (H). Arrow shows position of supercoil, bracket shows RNA:DNA hybrids. Middle, chemiluminescent imaging of each transcription reaction applied to hybridization membrane by dot blot and probed with S9.6 antibody and HRP-conjugated secondary antibody. Bottom, UV image of same dot blots stained with Ethidium Bromide.

## Description

Inactivation of the *PKD1* gene is responsible for most cases of Autosomal Dominant Polycystic Kidney Disease (ADPKD) (Paul & Vanden Heuvel, 2014) . ADPKD appears in adulthood in individuals with a single pathogenic *PKD1* allele, culminating in the development of fluid-filled cysts that can number in the thousands. Cysts form when the levels of the human *PKD1* gene product, polycystin 1, drop below a threshold that otherwise prevents cell proliferation (Luo et al., 2023; Ong & Harris, 2015) . Experimental evidence up to now largely agrees with a “two hit” bi-allelic inactivation model, where a cyst arises from a focal *PKD1* somatic inactivation event, although haploinsufficiency may also contribute (Paul & Vanden Heuvel, 2014; Qiu et al., 2023) . Regardless, molecular regulators of *PKD1* gene expression and stability are not fully defined. Previously, pyrimidine/purine-rich sequences in *PKD1* have been identified that block replication and activate DNA damage responses (Bacolla et al., 2001; Blaszak et al., 1999; Germino et al., 1992; Liu et al., 2012; Patel et al., 2004; Rider et al., 2022; Van Raay et al., 1996) . Here, we tested whether two *PKD1* sequences with a G/C sequence bias can form R-loops *in vitro* , which would suggest roles for this structure in *PKD1* regulation and stability.

R-loops are three-stranded structures that form when newly synthesized RNA pairs with the DNA template, thereby displacing the non-template strand for up to a kilobase. Although involved in gene expression, unresolved R-loops increase the risk of site-specific genetic instability (reviewed by (Niehrs & Luke, 2020; Petermann et al., 2022) ). A framework for understanding R-loop sequences came from analyses of immunoglobulin switch region R-loops (Daniels & Lieber, 1995; Duquette et al., 2004; Mizuta et al., 2003; Reaban et al., 1994; Reaban & Griffin, 1990; Tian & Alt, 2000; Yu et al., 2003) , which likely serve as obligate intermediates in the class switch recombination reaction (Maul et al., 2017) . A cytosine bias on the transcribed strand stabilizes RNA:DNA hybrids during transcription, and in the genome R-loops are now known to form at CpG islands, transcription terminator regions (Ginno et al., 2012, 2013) , repetitive elements (Gambelli et al., 2023) , and at telomeres (Toubiana & Selig, 2018) . Despite roles in gene expression, chromosome organization and dynamics (Gambelli et al., 2023; Hegazy et al., 2020; MacKay et al., 2020; Sanz et al., 2016) , R-loops are also a source of genome instability, making their regulation critical for avoiding conflicts between transcription and replication (Gambelli et al., 2023; Petermann et al., 2022) .

*PKD1* has a skewed distribution of G and C nucleotides, suggesting that R-loops may form in some regions upon transcription. With respect to the first kilobase of the coding sequence, *PKD1* is C-rich (~42%) on the transcription template (antisense strand). Previous studies have also demonstrated pyrimidine/purine repeats throughout introns 21 and 22 that interfere with replication activities (Bacolla et al., 2001; Liu et al., 2012; Patel et al., 2004; Rider et al., 2022) , and intron 22 indeed has a C-bias on the sense strand (~56%) (Van Raay et al., 1996) . Therefore, we selected 822 nucleotides of exon1/intron 1 and 704 nucleotides containing intron 22 to test for R-loop formation ( Figure 1A ). Both regions were cloned (Genscript) into pBluescript II SK ^+^ between the T7 and T3 promoters (named pBS_h *PKD1* _5’ and pBS_h *PKD1* _IVS22) ( Figure 1B ). *PKD1* sequences were orientated such that T3 transcription templates on the *PKD1* antisense strand for both clones, which is C-rich for the 5’ region and G-rich for IVS22. RNA:DNA hybridization is favored when the transcription template is C-rich, predicting R-loop formation for pBS_h *PKD1* _5’ upon T3 transcription, and for pBS_h *PKD1* _IVS22 upon T7 transcription.

Transcription of pBS_h *PKD1* _5’ with T3 polymerase, followed by resolution of the products by agarose electrophoresis, resulted in the generation of slower migrating species compared to supercoiled input DNA ( Figure 1C, top ). T7 expression or RNase H treatment, which degrades RNA:DNA hybrids, resulted in products that co-migrate with supercoiled DNA. Similarly, transcription of pBS_h *PKD1* _IVS22 with T7 RNA polymerase resulted in slower migrating DNAs compared to supercoiled input, and they reverted to supercoils upon RNase H treatment ( Figure 1C ). T3-directed expression of pBS_h *PKD1* _IVS22 did not significantly alter electrophoretic mobility compared to supercoiled input ( Figure 1C ).

To further test for RNA:DNA hybrid formation, we performed dot blot assays of the same transcription reactions using an R-loop-specific antibody (S9.6) (Boguslawski et al., 1986) , which has an affinity for RNA:DNA hybrids comparable to that of RNase H (Bou-Nader et al., 2022) . Probing the blots with S9.6 resulted in signals for T3 transcription of pBS_h *PKD1* _5’ and T7 transcription of pBS_h *PKD1* _IVS22 ( Figure 1 C, middle ). S9.6 signals were not observed for DNAs that co-migrated with supercoils ( Figure 1 C, middle ). Subsequent ethidium bromide staining and UV imaging of the dot blots showed DNA in all wells ( Figure 1C, bottom ). Collectively, we conclude that the 5’ region of *PKD1* supports co-transcriptional R-loop formation when transcribed in the sense orientation *in vitro* , while intron 22 supports R-loops when transcribed in the opposite orientation (antisense).

The ability of *PKD1* sequences to adopt co-transcriptional R-loops rationalizes a role for these structures in the regulation and/or stability of the gene, so it will be important to measure R-loops at the endogenous locus and identify how they contribute to *PKD1* regulation. C-rich DNA on the transcribed strand generally supports R-loops, and based on the sequence of human *PKD1* it is likely that there are additional R-loops sequences, yet roles in *PKD1* function remain untested. Interestingly, while we predict 5’-localized R-loop formation during transcription of *PKD1* , antisense or convergent transcription would be required for R-loop formation in introns 21 and 22. The presence of least one antisense lncRNA transcript ( *PKD1* -AS1) downstream of intron 22, raises the prospect of stable and internal R-loops in *PKD1* , the impact of which is unknown. However, the persistent occurrence of unscheduled R-loops in the genome increases the risk of mutagenesis (Gambelli et al., 2023; MacKay et al., 2020; Petermann et al., 2022) ; therefore, further characterization of non-B-form DNAs in *PKD1* will better define the molecular regulators of *PKD1* expression and mutagenesis that are relevant to ADPKD.

## Methods

**Clones**

Sequences (Extended Data) were synthesized and cloned by Genscript into pBluescript II SK ^+^ (Stratagene/Agilent, 212205) at EcoRI/HindIII sites. *PKD1* inserts were positioned between T7 and T3 promoters. For pBS_h *PKD1* _5’, 822 nts pf *PKD1* Exon 1 and the 5’ region of intron 1 was cloned. For pBS_h *PKD1* _IVS22 the entirety of intron 22, 704 nucleotides, was cloned. Plasmids were transformed into NEB Stable (New England Biolabs, C3040), grown overnight in liquid LB culture under ampicillin selection, and plasmid purified using Monarch miniprep kit (New England Biolabs, T1010). Plasmids were checked for purity by spectrophotometry and verified with agarose gel electrophoresis followed by sequencing of the insert region (Genewiz) using T3 and T7 primers.

**Transcription reactions**

Each 20-microliter transcription reaction included 400 ng of plasmid, 0.5 mM each rNTP (New England Biolabs, N0466S), 1 X manufacturer buffer, and 100 units of the indicated RNA polymerase, incubated at 37 °C for 1 hour. All reactions were treated with RNase A (50 ng/µl), and reactions with RNase H (5 units) included 1 X RNase H buffer and were incubated for 30 minutes at 37 °C. Transcription products were resolved by 1% agarose electrophoresis in 1X TAE buffer, post-stained with ethidium bromide, and imaged using an UVP GelSolo imaging system (Analytikjena). A representative image is shown from greater than 5 independent experiments.

**Dot Blot**

Transcription reactions were serially diluted and applied to Hybond nylon membrane (Amersham Biosciences, RPN303B) using a vacuum dot blot apparatus, washed with TE buffer, cross-linked by exposure to UV transilluminator for 30 seconds, and then blocked with TBS (50 mM Tris pH 7.5, 50 mM KCl) containing 5% milk for 2 h. The membrane was incubated with S9.6 antibody (Sigma, MABE1095, 1:1000) in TBS overnight. The following day, the membrane was washed three times with TBS, followed by incubation for 1 h with HRP-conjugated rabbit anti-mouse IgG (Invitrogen, 31457) diluted 1/3000 in TBS. After 3 washes, the membrane was incubated with 2 mL of WesternSure premium chemiluminescent substrate (Li-COR, 926-95000) for 5 min. Luminescence was then collected with a Li-COR Western Blot imager. A representative image is shown from more than 5 independent experiments.

## Reagents

**Table:** 

Enzyme	Description	Source
T7 RNA polymerase, 50,000 U/ml	Transcription from T7 promoter	New England Biolabs, M0251S
T3 RNA polymerase, 50,000 U/ml	Transcription from T3 promoter	New England Biolabs, M0378S
RNase A, 20 mg/ml	Ribonuclease specific for pyrimidines	New England Biolabs, T3018L
RNase H, 5,000 U/ml	Ribonuclease specific for RNA hybridized with DNA	New England Biolabs, M0297S

**Table:** 

Plasmid	Description	Source
pBS_h *PKD1* _5’	pBluescript II SK ^+^ with 822 nts of human *PKD1* exon/intron 1	GenScript
pBS_h *PKD1* _IVS22	pBluescript II SK ^+^ with 704 nts of human *PKD1* intron 22	GenScript

**Table:** 

Antibody	Description	Source
S9.6	Anti-RNA:DNA hybrid antibody	Sigma, MABE1095
HRP-conjugated rabbit anti-mouse IgG	Secondary antibody for detection of mouse IgG	Invitrogen, 31457

## Extended Data

Description: PKD1 sequences. Resource Type: Text. DOI: 10.22002/sqjps-qwd45
